# Minimally invasive surgery for pedal digital deformity: an audit of complications using national benchmark indicators

**DOI:** 10.1186/s13047-015-0073-x

**Published:** 2015-04-21

**Authors:** Mark Gilheany, Omar Baarini, Dean Samaras

**Affiliations:** East Melbourne Podiatry, Suite 4, Level 2, 182 Victoria Parade, Melbourne, VIC 3002 Australia; Australasian College of Podiatric Surgeons, PO BOX 248, Collins Street West, Melbourne, VIC 8007 Australia

**Keywords:** Clinical audit, Minimally invasive surgery, Percutaneous, Hammertoe, Complications

## Abstract

**Background:**

There is increasing global interest and performance of minimally invasive foot surgery (MIS) however, limited evidence is available in relation to complications associated with MIS for digital deformity correction. The aim of this prospective audit is to report the surgical and medical complications following MIS for digital deformity against standardised clinical indicators.

**Methods:**

A prospective clinical audit of 179 patients who underwent MIS to reduce simple and complex digital deformities was conducted between June 2011 and June 2013. All patients were followed up to a minimum of 12 months post operatively. Data was collected according to a modified version of the Australian Council of Healthcare standards (ACHS) clinical indicator program. The audit was conducted in accordance with the National Research Ethics Service (NRES) guidelines on clinical audit.

**Results:**

The surgical complications included 1 superficial infection (0.53%) and 2 under-corrected digits (0.67%), which required revision surgery. Two patients who underwent isolated complex digital corrections had pain due to delayed union (0.7%), which resolved by 6 months post-op. No neurovascular compromise and no medical complications were encountered. The results compare favourably to rates reported in the literature for open reduction of digital deformity.

**Conclusion:**

This audit has illustrated that performing MIS to address simple and complex digital deformity results in low complication rates compared to published standards. MIS procedures were safely performed in a range of clinical settings, on varying degrees of digital deformity and on a wide range of ages and health profiles. Further studies investigating the effectiveness of these techniques are warranted and should evaluate long term patient reported outcome measures, as well as developing treatment algorithms to guide clinical decision making.

## Background

There is increasing global interest and performance of minimally invasive foot surgery, reflected in recent publications within the peer review foot and ankle surgical literature [1-8]. MIS has been defined as the performance of osseous and soft tissue procedures through the smallest possible working incision without direct visualization of the deeper structures [2]. The techniques, which have been well described in published scientific literature, can be applied to reduce or correct common forefoot pathology including but not limited to hallux valgus, tailor’s bunionette deformity, central metatarsal pathology and lesser toe deformities [1-8].

The apparent benefits of MIS approaches compared to traditional open methods for foot and ankle surgery are promoted as including sparing of soft tissue, reduced post-operative pain, better cosmetic outcomes, shorter operating times, shorter hospital stay, reduced cost relating to time of surgery and surgical consumables/implants, reduced scarring and reduced risk of infection [2-8].

Historically, concerns regarding the potential for inadvertent tissue damage during MIS procedures have been raised [3]. Such concerns are reasonable as important neurovascular structures are in close proximity to the target tissues undergoing reconstruction. In open procedures these structures are visualised and can be directly protected. A cadaveric study designed to investigate the risk of neurovascular and tendon injury associated with MIS techniques in the forefoot demonstrated low risk to these structures [5]. However, no peer reviewed journal publications were identified by the authors as having investigated the safety of MIS techniques for the correction of digital deformities in a clinical setting.

The aim of this prospective audit is to report the surgical and medical complications following MIS for digital deformity against standardised clinical indicators.

## Methods

### Audit design

The study methods described are consistent with the principles of audit activity as defined by the National Research Ethics Service (NRES) [9]. The patients included in this audit were not allocated to specific treatment groups. Patients elected to have the procedures performed on the basis of guidance from the primary surgeon (MG) on the various surgical and non-surgical options available for the presenting conditions. The choice of open versus percutaneous techniques was not influenced by severity of deformity. The perioperative management protocols did not deviate from routine practice. This work complies with the ethics in publication policy of the Australasian College of Podiatric Surgeons. Consent was obtained from all patients included in the audit.

### Inclusion criteria

All patients who underwent minimally invasive procedures by a single surgeon (MG) for lesser toe pathology (digital deformity) over a two year period (June 2011 until June 2013) were included. This included patients who underwent multiple procedures for digital deformity as well as concomitant procedures such as hallux valgus or limitus/rigidus. All patients were followed for a minimum of 12 months post operatively.

### Perioperative management

The procedures were performed either under local anaesthetic (LA) or general anaesthetic (GA) with LA. All procedures were performed either in an office procedure room, hospital setting (including surgi centre) on an ambulatory day case basis.

Patients who underwent procedures within a hospital environment were administered intravenous antibiotic prophylaxis pre-operatively and a single subcutaneous dose of enoxaparin sodium 20 or 40 mg intra-operatively for thromboprophylaxis as part of routine protocol, often due to additional procedures involving the use of internal fixation (eg for hallux valgus correction). Those performed in the office setting did not receive either form of prophylaxis. All patients were managed with routine multimodal post-operative analgesic medication. Periodic icing and elevation of the affected limb(s) was also recommended for the first 48 hours.

### Instrumentation

All procedures were carried out using standard MIS hand instrumentation and Osada low speed/high torque power instrumentation. Fluoroscopy was utilised as appropriate with a Hologic InSight2 Fluoroscan Mini C-arm.

### Post-operative management

The post-operative dressing regimen consisted of routine compression dressings to the operated foot/feet incorporating digital splinting. The splinting was used to maintain desired alignment for up to 6 weeks post procedure, followed by return to capacious footwear.

Patients were reviewed in the clinical rooms within 7 days post operatively then again at 3, 6, 9, 12, 26 and 52 weeks. This regimen is reflective of the typical post-operative follow up performed by the primary author and his peers in Australia. All three authors, at various times, were involved in patient review and data collection as per the ACPS audit guidelines.

### Data collection

Vascular status was assessed immediately post operatively using clinical signs of colour and temperature as well as superficial venous plexus filling time (SVPFT). Neurological status was assessed utilising a Semmes-Weinstein 5.07/10 g monofilament at initial review and 6 weeks post operatively. Neurological status could not be assessed immediately post operatively due to the use of long acting local anaesthesia. Patients were assessed for signs of pedal infection at initial review and at subsequent review consultations.

General demographic data was collected from patient charts. The Patient American Society of Anesthesiologists (ASA) Score was also recorded given it is a global score to assess the physical status of patients before surgery [10].

Complications were recorded according to the clinical indicators as administered by the Australasian College of Podiatric Surgeons (ACPS) (Table 1). These indicators have been developed and validated utilising the ACHS clinical indicator program [11]. Complications related to the indicators were entered into the ACPS database as part of compliance with national audit.

**Table 1:** **ACPS surgical audit indicators**

	**Clinical indicators**
1	Infection (outpatient treatment within 30 days) Forefoot-superficial
2	Infection (outpatient treatment within 30 days) Forefoot- deep
3	Non-union requiring readmission within 9 months
4	Wound breakdown (outpatient within 30 days)
5	Wound breakdown (re-admission within 30 days)
6	Painful internal fixation device (readmission within 30 days)
7	Medical admission (chest pain, diabetes etc. related to podiatric admission within 7 days)
8	Reoccurrence of deformity requiring readmission within 30 days.
9	Over correction (requiring readmission within 30 days)
10	Under correction (requiring readmission within 30 days)
11	DVT (Outpatient treatment within 30 days)
12	DVT (requiring readmission within 30 days)
13	Pulmonary embolus (requiring readmission within 30 days)
14	Complex regional pain syndrome (within 30 days)
15	Other complication within 30 days

### Digital pathology definitions

**Figure 1:**
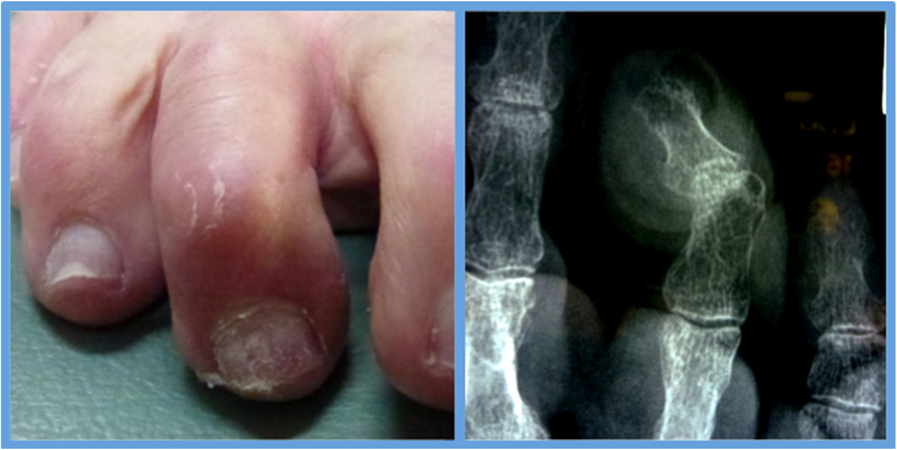
Simple digital deformity (pre-operative). Flexion deformity is seen at the distal interphalangeal joint of the right 3^rd^ toe clinically and radiographically.

Lesser toe pathology was defined as digital deformity resulting in a condition or malposition of the toe(s), which required surgical intervention. Digital deformity was further classified as simple or complex based upon the level of anatomic involvement. To clarify, the following definitions were applied:

*Simple* digital deformity included pathology, which was confined to the phalanges and soft tissue structures involving the interphalangeal joints (Figure 1). Surgical procedures in this group included percutaneous phalangeal osteotomies and/or osteectomy with or without percutaneous lengthening/release procedures to flexor/extensor tendons and capsular releases as required.

**Figure 2:**
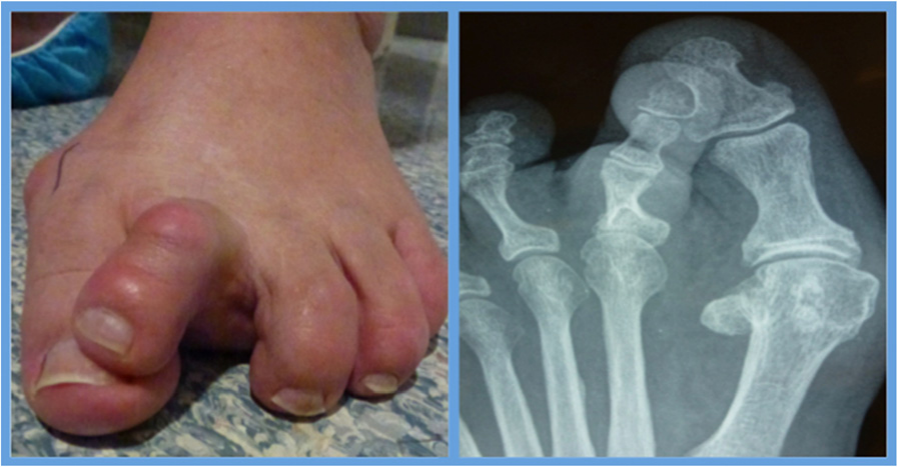
Complex digital deformity (pre-operative). Crossover 2^nd^ toe deformity with subluxation at the MTPJ as seen clinically and radiographically. Note the concomitant hallux valgus deformity.

*Complex* digital deformity included simple digital deformity with the addition of metatarsophalangeal joint (MTPJ) contracture resulting in subluxation or dislocation at that level (Figure 2). Surgical procedures in this group involved those described for simple deformity as well as percutaneous reconstructive procedures at the metatarsophalangeal joint level. Examples include distal metatarsal osteotomies and soft tissue releases at the MTPJ.

The procedures performed were based on the principles and techniques described in Maffulli and Easly [3].

## Results

A total of 179 patients (15 male, 164 female) underwent MIS surgery for digital deformity during this period. The age ranged between 15–93 years (mean 62 years) with a standard deviation of 8.25 years. 81% were 51 years of age or older.

Within the cohort, 124 patients were categorised as ASA 1 (69.3%), 54 ASA 2 (30.2%) and 1 patient ASA 3 (0.55%). The majority of comorbidities reported were controlled hypertension, hyperlipidaemia, asthma, osteoporosis and osteoarthritis. Five patients had Diabetes Mellitus (DM). Three patients had Type 1 DM and two had Type 2 DM. The latter two patients had a history of apical ulceration affecting the digit undergoing surgery. Five patients disclosed a history of smoking (tobacco).

Fifty five (55%) of cases were performed in a hospital setting and 45% were performed in an in office clinical procedure room. Sixty four (64%) were performed under general anaesthesia and local anaesthesia, whilst 36% were performed under a regional local anaesthetic alone. Local blocks were performed using either 0.75% ropivacaine hydrochloride or 0.5% bupivacaine hydrochloride (plain solution).

A total of 299 digits were operated on out of the 179 patients included in the study. Of these, 84 patients underwent surgery on multiple digits. Table 2 lists the number of simple and complex procedures performed and the associated adverse outcomes as they related to the ACHS clinical indicators.

**Table 2:** **Distribution of procedures and adverse outcomes**

		**Adverse outcomes**		
	Number of procedures performed	ACHS indicator 1 (superficial infection)	ACHS indicator 10 (undercorrection)	ACHS indicators 2,3,4,5,6,7,8,9,11,12,13,14,15 (Refer to Table 1 for descriptions)
Simple	203	0	1	0
Complex	96	1	1	0
**Totals**	**299**	**1**	**2**	**0**

Studies have reported a complication rate of up to 50% associated with traditional open digital surgery [12-14]. Most commonly pain, infection, neurovascular compromise, delayed healing, bony non-union, post operative wound infection and vascular impairment (requiring Kirschner wire removal) and medical complications such as deep vein thrombosis (DVT) [15-19]. The results of this audit indicate significantly lower complication rates with MIS techniques.

Superficial surgical site infection developed in one patient, which resolved following a single course of oral antibiotics. This represented a total infection rate of 0.56%, which is lower than averages reported in the literature for foot and ankle surgery [20,21]. This patient had well controlled Type II DM, the procedure was performed in a hospital setting and antibiotic prophylaxis was administered at the time of surgery. The diabetic population in this study represented just 2.8% of the cohort. Higher rates of infection would have been expected with a greater percentage of patients with DM particularly if complicated by peripheral neuropathy [21].

The under correction rate in this audit was 0.67% which may be further broken down into simple (0.33%) and complex (0.33%). Revision MIS procedures were performed 3 months post the initial surgery to improve alignment. Revision osteotomies were performed as well as appropriate soft tissue release to achieve rectus alignment. The post-operative management was as described in the methods section. The revision rates in this audit compare favourably to those of open digital deformity correction, which have been reported to range between 2–7.9% [19-22].

## Discussion

Many papers investigating MIS techniques report the reduced potential for neurovascular complications [3-6]. No patients in this audit developed neurological or vascular compromise. This is consistent with previous anecdotal claims that MIS techniques are associated with fewer iatrogenic complications, most notably involving the skin and vessels [23]. No blanching or cyanosis was exhibited in the immediate post operative period following MIS which does occur to varying degrees following open surgery and Kirschner wire fixation.

In 1991, White theorised that there would be less post operative pain/discomfort following MIS due to reduced dissection of soft tissue [6]. Although as part of our study we did not specifically assess post-operative pain levels, during clinical review 2 patients (0.7%) were identified as having persistent pain at 4 months post op. This was due to delayed healing of the osteotomy sites, which resolved by the 6 month review. They remained pain free at 12 month follow up.

The inclusion of variations in comorbidity demonstrated by the ASA and inclusion of patients with diabetes mellitus and smokers was anticipated to result in higher complication rates. In this cohort, comorbidities did not translate to increased complication rates. Furthermore there was no increase in complications with the performance of additional procedures required to reduce complicated deformities in the digits. Intraoperative conversion to open repair was not required in this cohort despite correction of complex digital deformities such as overlapping and crossover toes. Inclusion of patients of advanced age did not adversely impact the complication rates; those over the age of 75 represented 21% of the total population.

In this study 45% of all procedures were performed in an office based procedure room without fluoroscopic guidance. Whilst intra-operative image intensification can enhance the surgeon’s capability to perform these techniques it is not a substitute for surgeon experience [4]. Appreciation for traditional surgical approaches and three-dimensional anatomy are paramount when attempting digital deformity correction with MIS techniques [3-6] particularly in the absence of fluoroscopy. The authors advocate structured training in MIS techniques of the foot and ankle after a sound background and experience with open surgical techniques. The primary surgeons’ experience is greater than 20 years with open approaches and had completed structured education in MIS techniques prior to the audit process.

Despite the limitations of this audit, promising results have been illustrated in relation to the fundamental safety of performing minimal incision surgery in the digits.

The authors advocate future research to evaluate the nature and extent of digital surgery that can be performed via MIS techniques. Ideally such research should be prospective, long term and comparative to open techniques. It should include important clinical information such as surgeon learning curve, pre- and post-operative clinical outcome data using validated tools, union rates with radiographic data, pre and post-operative clinical images and a full cost benefit analysis. Ultimately the development of validated treatment algorithms is required in order to guide clinical decision making.

## Conclusion

This audit has illustrated that performing MIS to address simple and complex digital deformity results in low complication rates. The results compare favourably to rates reported in the literature for open reduction of digital deformity. MIS procedures were safely performed in a range of clinical settings, on varying degrees of digital deformity and on a wide range of ages and health profiles. Further studies investigating the effectiveness of these techniques are warranted and should evaluate long term patient reported outcome measures, as well developing treatment algorithms to guide clinical decision making.

## Abbreviations

MIS: Minimally invasive surgery
ACHS: Australian Council of Healthcare Standards
NRES: National Research Ethics Service
LA: Local anaesthesia
GA: General anaesthesia
SVPFT: Superficial venous plexus filling time
ASA: American Society of Anesthesiologists
ACPS: Australasian College of Podiatric Surgeons
MTPJ: Metatarsophalangeal joint
DM: Diabetes mellitus
DVT: Deep vein thrombosis
